# Effects of primary granulocyte colony-stimulating factor (G-CSF) prophylaxis for chemotherapy-induced febrile neutropenia in diffuse large B-cell lymphoma patients receiving the R-CHOP-21 regimen

**DOI:** 10.2478/abm-2025-0021

**Published:** 2025-09-02

**Authors:** Pannathorn Nakaphan, Noppacharn Uaprasert

**Affiliations:** Faculty of Medicine, Chulalongkorn University, Bangkok 10330, Thailand; Division of Hematology, Department of Medicine, Faculty of Medicine, Chulalongkorn University and King Chulalongkorn Memorial Hospital, Thai Red Cross Society, Bangkok 10330, Thailand; Center of Excellence in Translational Hematology, Faculty of Medicine, Chulalongkorn University and King Chulalongkorn Memorial Hospital, Thai Red Cross Society, Bangkok 10330, Thailand

**Keywords:** diffuse large B-cell lymphoma, febrile neutropenia, G-CSF, primary prophylaxis, R-CHOP

## Abstract

**Background:**

Primary prophylaxis with granulocyte colony-stimulating factor (G-CSF) in diffuse large B-cell lymphoma (DLBCL) patients undergoing rituximab-cyclophosphamide-doxorubicin-vincristine-prednisolone every 21 d (R-CHOP-21) chemotherapy varies based on physician discretion.

**Objectives:**

The present study aims to investigate the impact of primary G-CSF prophylaxis on febrile neutropenia (FN) and other outcomes in DLBCL patients receiving R-CHOP-21 in real-world practice.

**Methods:**

Medical records of 103 newly diagnosed DLBCL patients, aged 18–80 years, were retrospectively analyzed. Seventy-four patients received primary G-CSF prophylaxis (prophylaxis group), while 29 patients did not receive prophylaxis (non-prophylaxis group). The occurrence of FN and other outcomes was compared between the two groups.

**Results:**

The prophylaxis group had older patients (median ± interquartile ranges [IQR], 63 ± 12 years vs. 50 ± 15 years, *P* < 0.001). The incidence of FN after the first R-CHOP was not statistically significant between groups (8.3% vs. 17.2%, *P* = 0.177). However, FN events were significantly higher in non-prophylaxis cycles (7.40%) compared with prophylaxis cycles (2.70%) (*P* = 0.027). Cumulative FN events were lower in the prophylaxis group (14.9%) than in the non-prophylaxis group (27.6%) (*P* = 0.134). FN-free survival was not significantly different between prophylaxis and non-prophylaxis groups (hazard ratio [HR], 0.48; 95% confidence interval [95%CI], 0.17–1.35), while primary G-CSF prophylaxis significantly improved event-free survival (HR, 0.36; 95%CI, 0.16–0.84).

**Conclusions:**

Primary G-CSF prophylaxis may reduce the risk of FN in DLBCL patients undergoing R-CHOP-21 treatment. The present study highlights the importance of primary G-CSF prophylaxis, especially for those at high risk of FN.

Diffuse large B-cell lymphoma (DLBCL) is an aggressive B-cell neoplasm and is the most common lymphoma subtype encountered in clinical practice. Rituximab plus cyclophosphamide, doxorubicin, vincristine, and prednisolone administered every 21 d (R-CHOP-21) is the standard treatment for DLBCL and can cure 60%–70% of patients [1,2,3,4]. However, patients receiving R-CHOP are at risk for potentially fatal chemotherapy-induced febrile neutropenia (FN).

Several meta-analyses have shown that primary prophylaxis with granulocyte colony-stimulating factor (G-CSF) can reduce the risk of FN by up to 50% in patients with solid cancers and malignant lymphomas [5,6,7,8]. Most guidelines recommend G-CSF prophylaxis if the risk of FN is ≥20% for all planned cycles of chemotherapy regimens. However, for R-CHOP-21, which is typically classified as an intermediate-risk regimen with a 10%–20% risk of FN according to most guidelines, physicians must evaluate individual patient factors associated with FN risk to determine whether primary G-CSF prophylaxis is necessary [9,10,11]. This evaluation often leads to variable use of primary G-CSF prophylaxis in real clinical practice.

Despite up to half of patients receiving primary G-CSF prophylaxis, the incidence of FN after R-CHOP-21 ranges from 13% to 41% [12,13,14,15,16]. This suggests that the risk of FN without primary G-CSF prophylaxis exceeds 20%, and routine primary G-CSF prophylaxis should be considered in general practice for R-CHOP-21. Additionally, factors associated with FN after R-CHOP-21 should be determined for patients with and without primary G-CSF prophylaxis.

The present study aims to determine the prevalence of FN after R-CHOP-21 in patients with newly diagnosed DLBCL and to assess the impact of primary prophylaxis with G-CSF on FN, as well as other clinical outcomes such as septic shock, death, and dose reduction. Furthermore, the present study aims to identify other factors associated with the development of FN after R-CHOP-21.

## Method

The present study was performed in accordance with the principles of the Declaration of Helsinki. Approval was granted by the ethics committee of the Faculty of Medicine, Chulalongkorn University (COA No. 1099/2021). Informed consent was waived due to the retrospective and observational nature of the study.

### Study design

We retrospectively reviewed the medical records of newly diagnosed patients with DLBCL aged between 18 years and 80 years, who received R-CHOP-21 treatment at King Chulalongkorn Memorial Hospital between 2018 and 2020. Patients treated with R-mini-CHOP (doxorubicin <35 mg/m^2^) in the first cycle, those with baseline absolute neutrophil counts (ANC) <1,000/μL, or baseline platelet counts <50,000/μL were excluded from the study. The standard protocol for newly diagnosed DLBCL at our institution is R-CHOP-21 at the standard dose for 6 cycles. The use of G-CSF was at the discretion of the attending physicians. G-CSF, administered as a 300 μg subcutaneous injection daily, was given for 7 d starting 1–3 d after R-CHOP-21, according to the institute's protocol. For patients receiving pegylated G-CSF, the original drug Neulastim (Amgen Manufacturing), administered as a single 6 mg sub-cutaneous injection, was given 1–3 d after R-CHOP-21. The choice between original or biosimilar G-CSF and pegylated G-CSF was based on the discretion of the attending physicians.

Varicella zoster reactivation prophylaxis using acyclovir and *Pneumocystis jirovecii* pneumonia prophylaxis using sulfamethoxazole-trimethoprim were routinely administered to patients receiving R-CHOP-21 if there was no history of allergy to these medications.

Central nervous system (CNS) prophylaxis using intrathecal methotrexate (12.5–15 mg), administered on the same day as R-CHOP-21, or intravenous high-dose methotrexate (3–4 g/m^2^) for 2 cycles, administered after the complete treatment of R-CHOP-21, was at the discretion of the attending physicians, primarily based on the sites of extranodal involvement.

### Outcomes

The primary objective was to compare the incidence of FN after the first R-CHOP cycle between patients who received primary G-CSF prophylaxis and those who did not. Secondary objectives included the differences in cumulative FN events and the composite outcome (FN events, reactive G-CSF rescue, and discontinuation of chemotherapy) after all R-CHOP cycles. Additionally, we analyzed the FN-free and event-free survival (FN, reactive G-CSF rescue, and discontinuation of chemotherapy) and determined the differences in FN events between prophylaxis and non-prophylaxis cycles. We categorized prophylaxis cycles as those using primary G-CSF prophylaxis until the first FN event, and non-prophylaxis cycles as those without primary G-CSF prophylaxis. Cycles with reactive G-CSF administration were excluded from analysis. We also aimed to assess the risks associated with FN if there were sufficient data for analysis.

### Statistical analysis

We planned to include all patients receiving R-CHOP who were eligible according to the study criteria during the study period. However, to ensure a sufficient sample size, power calculations were conducted using the estimated prevalence of FN in patients receiving R-CHOP-21, which is 40%. We anticipated needing at least 93 patients, which corresponds to a 95% confidence interval (95% CI) and a degree of accuracy of 0.1 [17].

Statistical analysis was performed using SPSS version 25.0 for Windows (SPSS Inc.) and NCSS 2021 version 21.0.4 (NCSS, LLC). For descriptive statistics, normality of the data was tested using Shapiro–Wilk test. Continuous data were presented as means (±standard deviation [SD]) or medians with interquartile ranges (IQR) as appropriate. Continuous variables were compared using the independent sample *t*-test, while categorical variables were compared using Pearson's chi-square test. For selected outcomes with non-parametric data, the Mann–Whitney *U* test was used. Kaplan–Meier survival analysis was used to assess FN-free and event-free survival. Binary logistic regression was used to compute the odds ratios (ORs) of multiple risk factors for FN events.

## Results

### Patient characteristics

A total of 103 newly diagnosed patients with DLBCL were included for analysis, with 74 (71.8%) receiving primary G-CSF prophylaxis (prophylaxis group) and 29 (28.2%) not receiving primary G-CSF prophylaxis (non-prophylaxis group). Of the 74 patients in the prophylaxis group, 61 (82.4%) received biosimilar G-CSF, 3 (4.1%) received original G-CSF, and 10 (13.5%) received pegylated G-CSF. The baseline characteristics of the patients are shown in **Table 1**. Patients in the prophylaxis group were significantly older (median ± IQR, 63 ± 12 years) than those in the nonprophylaxis group (50 ± 15 years) (*P* < 0.001). Additionally, the prophylaxis group contained a higher proportion of patients aged >60 years than the non-prophylaxis group (72.5% vs. 13.8%, *P* < 0.001). Other baseline characteristics were similar between the two groups.

**Table 1.** Clinical and laboratory characteristics of patients with newly diagnosed DLBCL before first R-CHOP

	**Prophylaxis (N = 74)**	**Non-prophylaxis (N = 29)**	***P***
Age (years)†	63 (12)	50 (15)	<0.001
Male (%)	32 (43.2)	15 (51.7)	0.437
Body weight (kg)	60.1 (11.9)	61.7 (12.3)	0.53
Height (m)	1.61 (0.09)	1.62 (0.08)	0.463
BMI (kg/m^2^)	23.0 (3.4)	23.2 (4.1)	0.882
Hemoglobin (g/dL)	11.5 (2.5)	11.7 (2.3)	0.742
WBC count (×10^9^/L)	8.62 (4.61)	8.97 (5.79)	0.749
ANC (×10^9^/L)	6.19 (4.22)	5.84 (3.99)	0.697
AMC (×10^9^/L)	0.67 (0.52)	0.60 (0.53)	0.582
Platelet count (×10^9^/L)	313.6 (160.0)	330.2 (109.2)	0.608
LDH (U/L)	463.6 (493.5)	488.6 (430.3)	0.811
Creatinine (mg/dL)	0.80 (0.27)	0.71 (0.18)	0.089
AST (U/L)	36.0 (38.0)	33.2 (25.8)	0.717
ALT (U/L)	33.9 (46.3)	48.7 (76.8)	0.24
Serum albumin (g/dL)	3.8 (0.7)	3.9 (0.6)	0.363
Stage (1, 2, 3, 4)	9, 23, 12, 30	5, 11, 1, 12	0.336
ECOG (0, 1, 2, 3, 4)	20, 42, 9, 2, 0	11, 15, 1, 0, 0	0.341
IPI (L, LI, HI, H)	15, 16, 26, 17	8, 10, 9, 2	0.18
Bone marrow involvement (%)	9 (12.2)	3 (10.3)	0.779
Comorbidities (%)	37 (50)	8 (27.6)	0.529
HBV Ag positive (%)	3 (4.1)	4 (13.8)	0.071
Anti-HCV Ab positive‡ (%)	1 (1.4)	0 (0)	0.531
Anti-HIV Ab (%)	1 (1.4)	0 (0)	0.526
CNS prophylaxis (%)	26 (35.1)	10 (34.5)	1.00

† Median (interquartile range); other parameters are presented as mean (±SD).

‡ Confirmed with HCV viral load.

Ab, antibody; Ag, antigen; ALT, alanine aminotransferase; AMC, absolute monocyte count; ANC, absolute neutrophil count; AST, aspartate aminotransferase; BMI, body mass index; CNS, central nervous system; DLBCL, diffuse large B-cell lymphoma; HBV, hepatitis B virus; HCV, hepatitis C virus; HIV, human immunodeficiency virus; IPI, international prognostic index; LDH, lactate dehydrogenase; SD, standard deviation; WBC, white blood cell.

### FN after the first cycle of R-CHOP

Of the 103 DLBCL patients included in the study, 11 (10.7%) developed FN after the first cycle of treatment. Of the 74 patients in the prophylaxis group, 6 (8.3%) developed FN, while 5 of 29 (17.2%) patients without prophylaxis developed FN (*P* = 0.177). Furthermore, 10 (34.9%) patients in the non-prophylaxis group received reactive G-CSF treatment. Despite G-CSF rescue, 5 patients still developed FN. The laboratory results after the first chemotherapy cycle are shown in **Table 2**. The median time for laboratory tests was 7.4 d and 8.3 d in the non-prophylaxis and in the prophylaxis group, respectively. Patients in the prophylaxis group had significantly higher ANC (7.13 × 10^3^/μL vs. 4.26 × 10^3^/μL, *P* = 0.029) and absolute monocyte counts (AMC) (0.28 × 10^3^/μL vs. 0.07 × 10^3^/μL, *P* < 0.001) than those in the non-prophylaxis group.

**Table 2.** Clinical and laboratory characteristics of patients with newly diagnosed DLBCL after first R-CHOP

	**Prophylaxis (N = 74)**	**Non-prophylaxis (N = 29)**	***P***
FN	6	5	0.177
Neutropenia (%)	43 (58.1)	15 (51.7)	0.557
Grade 4 neutropenia (%)	18 (24.3)	7 (24.1)	0.98
G-CSF rescue (%)	NA	10 (34.5)	NA
Hemoglobin (g/dL)	11.4 (2.1)	11.0 (2.3)	0.501
WBC count (×10^9^/L)	8.29 (10.44)	5.88 (5.96)	0.245
ANC (×10^9^/L)	7.13 (9.97)	4.26 (3.07)	0.029
AMC (×10^9^/L)	0.28 (0.47)	0.07 (0.12)	<0.001
Platelet count (×10^9^/L)	221.6 (161.2)	287.8 (118.0)	0.047

AMC, absolute monocyte count; ANC, absolute neutrophil count; DLBCL, diffuse large B-cell lymphoma; FN, febrile neutropenia; G-CSF, granulocyte colony-stimulating factor; NA, not applicable; WBC, white blood cell.

### The outcomes of all treatment cycles

The results of the R-CHOP treatment cycles are summarized in **Table 3**. The cumulative FN events were 19 (18.4%), with the majority occurring after the first cycle (57.9% of cases). Although the non-prophylaxis group had a higher rate of cumulative FN events (27.6%) compared with the prophylaxis group (14.9%), this difference was not statistically significant (*P* = 0.134). Among the 19 cases of FN, organisms were identified in 4 events (*Escherichia coli*, *Klebsiella pneumoniae*, Salmonella group B, and *Enterococcus faecalis*).

**Table 3.** Clinical data, laboratory characteristics, and outcomes of patients with newly diagnosed DLBCL after all cycles of R-CHOP

	**Prophylaxis (N = 74)**	**Non-prophylaxis (N = 29)**	***P***
Total cycles received†	6 (0)	6 (0)	0.483
Total treatment duration(d)†	108.5 (14)	107 (8)	0.395
Relative dose intensity (%)	89.0 (14.2)	91.1 (9.7)	0.461
Complete remission (%)	52 (70.3)	19 (65.5)	0.639
FN (%)	11 (14.9)	8 (27.6)	0.134
Reactive G-CSF rescue (%)	NA	19 (65.5)	NA
Chemotherapy discontinuation (%)	6 (8.1)	3 (10.3)	0.718
Composite outcome (%)	15 (20.3)	14 (48.3)	0.004
Hospitalization (%)	16 (21.6)	8 (27.6)	0.567
ICU admission (%)	1 (1.4)	0	0.105

† Median (interquartile range).

DLBCL, diffuse large B-cell lymphoma; FN, febrile neutropenia; G-CSF, granulocyte colony-stimulating factor; ICU, intensive care unit; NA, not applicable.

In the non-prophylaxis group, 19 (65.50%) received reactive G-CSF rescue. Discontinuation of chemotherapy occurred in 3 (10.3%) and 6 (8.1%) in the non-prophylaxis and prophylaxis groups, respectively. The non-prophylaxis group had a significantly higher rate of composite events (48.3% vs. 20.3%, *P* = 0.004) compared with the prophylaxis group. However, there were no statistically significant differences between the groups in terms of total R-CHOP cycles, treatment duration, doxorubicin dose, or relative dose intensity.

**Figure 1.**
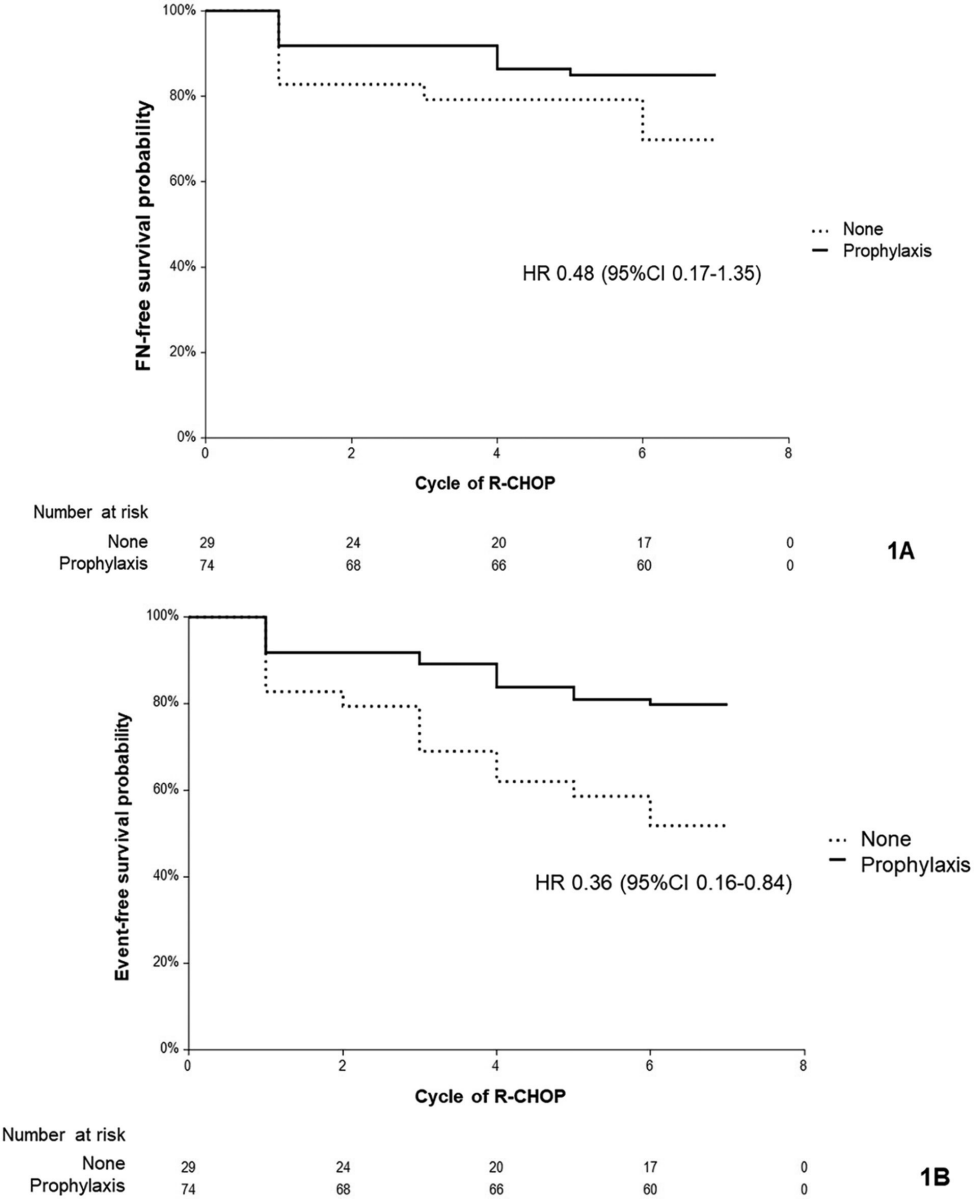
Survival analysis demonstrates FN-free survival probability **(A)** (HR = 0.48; 95% CI, 0.17–1.35) and event-free survival probability **(B)** (HR = 0.36; 95% CI, 0.16–0.84). 95% CI, 95% confidence interval; FN, febrile neutropenia; HR, hazard ratio.

Survival analysis revealed that FN-free survival was not significantly different between the prophylaxis and non-prophylaxis groups (hazard ratio [HR] = 0.48; 95% CI, 0.17–1.35) (**Figure 1A**). However, the prophylaxis group had a significantly better event-free survival (including FN, reactive G-CSF use, or chemotherapy discontinuation) than the non-prophylaxis group (HR = 0.36; 95% CI, 0.16–0.84) (**Figure 1B**).

### Individual cycle analysis

We conducted an analysis of FN events for each cycle of chemotherapy treatment and identified a total of 405 primary prophylaxis cycles and 94 non-prophylaxis cycles. FN events were significantly more frequent in the non-prophylaxis cycles (7.40%) compared with the prophylaxis cycles (2.70%) (*P* = 0.027).

### Univariate analysis for risks associated with FN

We performed binary logistic regression and found that hepatitis B virus (HBV) carrier status (OR, 7.02; 95% CI, 1.42–34.63) and CNS prophylaxis (OR, 3.25; 95% CI, 1.18–8.04) were the only factors significantly associated with FN (**Table 4**). Due to inadequate power, we did not perform multivariate analysis.

**Table 4.** Risks associated with FN after R-CHOP

		**Odd ratio (95% CI)**	***P***
Age (years)	≤35	Reference	
	>35–50	0.18 (0.01, 2.32)	0.187
	>50–65	0.63 (0.11, 3.72)	0.611
	>65	1.04 (0.17, 6.26)	0.963

Stage	1	Reference	
	2	1.26 (0.12, 13.25)	0.848
	3	5.78 (0.55, 60.61)	0.144
	4	4.61 (0.54, 39.49)	0.163

Bone marrow involvement	No	Reference	
	Yes	2.50 (0.67, 9.38)	0.174

HBV carrier	No	Reference	
	Yes	7.02 (1.42, 34.63)	0.017

CNS prophylaxis	None	Reference	
	Yes	3.25 (1.18, 8.04)	0.020

G-CSF prophylaxis	None	Reference	
	Yes	0.46 (0.17, 1.21)	0.118

95% CI, 95% confidence interval; CNS, central nervous system; FN, febrile neutropenia; G-CSF, granulocyte colony-stimulating factor; HBV, hepatitis B virus.

Among the tested patients, 7 (6.8%) were tested positive for HBs antigen (HBs Ag), with 4 in the non-prophylaxis group and 3 in the prophylaxis group. None of the HBV carriers had active hepatitis, and all received anti-HBV therapy, predominantly lamivudine. The baseline white blood cell (WBC) counts between HBV carriers and non-HBV carriers were 7.12 × 10^9^/L and 8.83 × 10^9^/L, respectively, which did not differ significantly (*P* = 0.383). Of the 7 HBV carriers, 4 (57.1%) developed FN; 3 had not received G-CSF prophylaxis, while 1 had received it.

Of the 36 patients receiving CNS prophylaxis, 26 (35.1%) received primary G-CSF prophylaxis, while 10 (34.5%) did not receive prophylaxis, which did not differ significantly (*P* = 1.000). The reasons for patient selection and the protocols used for CNS prophylaxis were not clearly specified in the medical records. There were 15 patients receiving intrathecal methotrexate, 17 receiving intravenous high-dose methotrexate, and 4 receiving both intrathecal and intravenous prophylaxis. Although the proportions of patients receiving CNS prophylaxis between the primary G-CSF prophylaxis and non-prophylaxis groups were not significantly different, CNS prophylaxis was associated with the risk of FN in patients treated with R-CHOP-21.

## Discussion

The use of primary G-CSF prophylaxis varies in R-CHOP-21, a chemotherapy regimen conventionally classified as intermediate FN risk, due to the need for physicians to assess the individual patient's risk. In the present study, approximately 70% of newly diagnosed DLBCL patients received primary G-CSF prophylaxis after R-CHOP treatment. This rate is higher than that reported in previous studies, where approximately half of the high-risk population did not receive primary G-CSF prophylaxis [12, 18]. However, despite the high rate of primary G-CSF prophylaxis usage in the present study, the rates of FN after the first cycle and the cumulative rate of FN were still high at 10.7% and 18.7%, respectively. Notably, the incidence of FN was 17.2% after the first cycle and 27.6% after all cycles in the non-prophylaxis group, where almost 90% of the patients were <60 years old. These findings suggest a higher incidence of FN in Thai patients with newly diagnosed DLBCL who received R-CHOP than what has been reported in randomized controlled trials or guidelines. A meta-analysis has shown that the rates of FN in observational studies were significantly higher than those reported by randomized controlled trials. Therefore, population-based studies are needed to determine the rates of FN in real-world practice before guidelines can be applied in clinical practice [19].

Approximately two-thirds of patients who did not receive primary prophylaxis required G-CSF administration during neutropenic periods. However, even with G-CSF rescue, FN still occurred in about half of these patients. The study findings indicate that primary G-CSF prophylaxis during the R-CHOP treatment phase is significantly more effective in preventing FN than reactive G-CSF rescue during the nadir neutropenic phase. Therefore, it is important for clinicians to consider primary G-CSF prophylaxis as a preventive measure, rather than relying on reactive rescue treatment, in order to minimize the risk of FN in patients undergoing R-CHOP treatment.

Previous studies have demonstrated a significant reduction in the incidence of FN with the primary use of G-CSF, particularly pegfilgrastim, in older age groups [20, 21]. In our cohort, which included patients aged 18–80 years old, primary G-CSF prophylaxis did not statistically decrease the rate of FN. However, it is important to note that patients in our prophylaxis group may have been considered at higher risk by their physician, as shown by the significant difference in age between the prophylaxis and non-prophylaxis groups. A previous retrospective study has also reported similar differences in baseline characteristics, such as disease stage, baseline laboratory values, and comorbidities, between the prophylactic and non-prophylactic groups [20]. The lack of difference in the development of FN, despite the prophylactic group being perceived as higher risk, could be interpreted as justifying the administration of G-CSF prophylaxis in this population.

Despite the lack of statistical significance in reducing the rate of FN in our cohort with primary G-CSF prophylaxis, the use of prophylaxis was still beneficial in reducing composite events during chemotherapy. Chemotherapy interruptions or dose reductions may lead to prolonged treatment duration and increased risk of disease progression or relapse. In fact, previous studies have demonstrated that primary G-CSF prophylaxis can help avoid dose intensity reductions. Therefore, the use of G-CSF prophylaxis in high-risk patients should be carefully considered, even if the reduction in FN rates may not reach statistical significance [20].

In the non-prophylaxis group, all patients who developed FN or received G-CSF rescue were subsequently given secondary G-CSF prophylaxis in the following cycles. To assess the impact of G-CSF on FN events, we conducted a sensitivity analysis on individual cycles. The results showed that G-CSF prophylaxis significantly reduced the total incidence of FN events. Additionally, 22 cycles of R-CHOP-21 treatment were required to prevent one case of FN through the use of G-CSF prophylaxis. These findings support the recommendation for routine primary G-CSF prophylaxis for patients with newly diagnosed DLBCL in Thailand who are treated with R-CHOP-21. Therefore, the use of primary G-CSF prophylaxis should be strongly considered to reduce the risk of FN and maintain treatment adherence.

Previous studies have identified several risk factors for the development of FN in patients receiving R-CHOP-21, including female sex, disease stage, international prognostic index (IPI), advanced age, poorer performance status, bone marrow involvement, anemia, and low baseline serum albumin [14, 18]. However, in our study, HBV carrier status and CNS prophylaxis were the only risk factors significantly associated with the development of FN.

Approximately half of the HBV carriers in our cohort developed FN, but the reasons for this higher risk are not yet fully understood. The prevalence of HBV carriers among adults in Thailand ranges from 3.78% to 5.99%, with most cases resulting from vertical transmission [22]. Although none of the HBV carriers in our study had active hepatitis B or overt cirrhosis, subclinical liver disease or liver fibrosis could not be completely ruled out. These factors may be linked to delayed hepatic clearance of certain chemotherapy agents, especially doxorubicin. Given these findings, we recommend primary G-CSF prophylaxis for HBV carriers receiving R-CHOP-21.

CNS prophylaxis with methotrexate is commonly used for DLBCL patients with factors suggesting a high risk of CNS relapse. However, the benefits of either intrathecal methotrexate or systemic high-dose methotrexate are controversial. Currently, there are no gold-standard guidelines for CNS prevention due to conflicting results among studies [23, 24]. The addition of intrathecal low-dose methotrexate to R-CHOP may increase the risk of neutropenia, resulting in an increased risk of FN [25]. Although half of the patients with CNS prophylaxis received separate systemic high-dose methotrexate after complete R-CHOP treatment, these patients might carry other factors associated with FN, which could not exclude these interactions in our study due to the insufficient power for multivariate analysis.

The interpretation of our study may be limited by the relatively small sample size, which may not allow us to demonstrate the statistical significance of G-CSF in preventing FN and detect other risks associated with R-CHOP-21-induced FN. However, other studies conducted in Western and Asian populations have reported rates of FN exceeding 20% in patients without G-CSF support. Furthermore, the use of G-CSF prophylaxis has been shown to reduce the risk of FN in real-world practice. The findings of our study are consistent with those of previous cohorts from different countries. Therefore, we recommend that primary G-CSF prophylaxis should be routinely offered to all patients receiving R-CHOP-21, especially those at higher risk.

## Conclusions

Our study highlights the high risk of FN among patients with DLBCL who receive R-CHOP-21, particularly those who do not receive G-CSF prophylaxis. Moreover, our findings suggest that HBV carriers and patients receiving CNS prophylaxis are at greater risk for FN. These results support the use of primary G-CSF prophylaxis as a routine measure for the treatment of newly diagnosed DLBCL patients treated with R-CHOP-21.
